# Impact of Climate on the Growth and Yield of the Main Tree Species from Romania Using Dendrochronological Data

**DOI:** 10.3390/plants14081234

**Published:** 2025-04-18

**Authors:** Marin Gheorghe, Bogdan M. Strimbu

**Affiliations:** 1 National Institute for Research and Development in Silviculture, National Forest Inventory, Bd. Eroilor 128, 077190 Voluntari, Romania; ghmarin@roifn.ro; 2 Faculty of Silviculture and Forest Engineering, Transilvania University of Brasov, Bd. Eroilor 29, 500036 Brașov, Romania; 3 National Institute for Research and Development for Biological Sciences, Splaiul Independenței 296, 060031 Bucharest, Romania; 4 College of Forestry, Oregon State University, 3100 Jefferson Way, Corvallis, OR 97333, USA

**Keywords:** ecoregions, increment cores, repeated measurements models, *Picea abies*, *Fagus sylvatica*, *Quercus petraea*

## Abstract

National Forest Inventories (NFIs) offer a comprehensive and consistent dataset for forest analysis, enabling the refinement of growth and yield models by integrating regional environmental factors. This study investigates the influence of climate on the growth of three dominant tree species in Romania: Norway spruce (*Picea abies* L. Karst), European beech (*Fagus sylvatica* L.), and Sessile oak (*Quercus petraea* (Matt.) Liebl). Increment core analysis revealed a general increase in diameter growth since 1960, partially correlated with temperature trends. Repeated measures analysis confirmed significant variations in radial growth across ecoregions. The analysis further explored the impact of climatic variables on diameter at breast height (DBH) and basal area (BA) growth and yield. Among nine climatic attributes and their combinations, total precipitation and average growing season temperature significantly affected DBH and BA growth. However, yield was largely insensitive to precipitation, with only Sessile oak yield showing a temperature dependence. Beyond ecoregion and climate, the growth and yield of DBH and BA exhibited positive correlations with the calendar year, age, and previous growth/yield values. Notably, DBH and BA growth demonstrated a dependence on the preceding four to five years, whereas yield was significantly influenced only by the previous year. The observed influence of both the calendar year and previous years suggests a prolonged environmental memory in tree growth and yield responses.

## 1. Introduction

While the third millennium witnessed rapid technological advancements, progress in forest modeling, particularly in regions relying on outdated approaches, lagged significantly [1]. These regions often suffer from a lack of empirical data [2] and robust, legally defensible quantitative methods [3]. Despite evidence advocating for the integration of spatial [4] and temporal components [5] into existing models, researchers frequently resort to questionable equations due to limited alternatives [6,7]. Although some researchers have initiated species-specific model development, progress remains slow due to insufficient support [4]. National Forest Inventories (NFIs) provide a crucial resource for representing forest dynamics, offering essential data for model development and calibration in countries lacking sophisticated modeling frameworks [8,9,10]. NFIs typically record species abundance and dimensional data [8,11], and may include supplementary information such as soil type and increment cores [12]. Repeated measurements of diameter at breast height (DBH), total height, and increment cores from NFIs form the basis for developing complex growth and yield models. However, substantial species variability across large geographic ranges often necessitates the delineation of homogeneous ecoregions prior to modeling.

Stratifying the study area is essential for enhancing the accuracy and precision of general growth and yield models [13,14,15,16]. While distinct model versions are expected for continental-scale countries like Canada and the USA [15], smaller countries with significant geomorphological and climatic heterogeneity, such as Romania (comparable in size to Oregon), also require differentiated modeling approaches. Marin et al. [4] demonstrated the need for distinct models or parameterizations for DBH growth in Romania. Based on over 6500 increment cores Marin et al. [4] delineated seven ecoregions for Norway spruce (*Picea abies* L. Karst), eight for Sessile oak (*Quercus petraea* (Matt.) Liebl.), and eleven for European beech (*Fagus sylvatica* L.).

Norway spruce, European beech, and Sessile oak, the three species selected by Marin et al. [4], are ecologically, socially, and economically dominant in Romania. They constitute 58% of the country’s forest area, 68% of its growing stock, and contribute two-thirds of wood-based revenue. Despite Norway spruce and European beech existing at the southeastern and eastern edges of their natural ranges, respectively, they exhibit vigorous growth in Romania. Sessile oak, well within its natural range, displays strong vitality and no apparent climate change impacts. Furthermore, over 70 years of data have been collected to model Romanian forest dynamics [17]. However, this extensive data collection has not been matched by commensurate modeling efforts; existing models lack independent validation and replication [3,18], and the absence of confidence intervals raises concerns about their performance [3,19].

While spatial variability—such as geographic location and local geomorphology—is a fundamental prerequisite for growth and yield modeling, changes in climatic factors can have compounding effects [20,21,22]. Although trees are long-lived organisms, decadal deviations from long-term averages of meteorological attributes, particularly temperature and precipitation, can significantly alter spatial findings [20,21,22]. This raises an important question: Are spatially localized growth and yield models sufficiently sensitive to capture the range of changes in tree development, or do multi-annual weather dynamics also play a significant role in influencing tree size? Therefore, this study aims to identify the climatic and geomorphological regions within Romania that require species-specific growth and yield relationships. The focus is on identifying and quantifying the impact of climate change on the annual growth of Norway spruce, European beech, and Sessile oak across Romanian ecoregions. These three species are not only significant from both economic and ecological perspectives but also thrive in distinctly different climates, which strengthens the potential for generalizing our findings. Specifically, we will investigate the relationships between annual and cumulative DBH and basal area (BA) growth and the primary meteorological variables recorded by the Romanian National Meteorological Administration.

## 2. Methods

The focal tree species of this study—Norway spruce, European beech, and Sessile oak—exhibit a distinct altitudinal distribution. Sessile oak dominates lower elevations, while Norway spruce occupies higher elevations, both forming pure and mixed stands. European beech occurs at intermediate altitudes, frequently coexisting with either Sessile oak or Norway spruce. The climate characterizing the three species varies not only with altitude but also with aspect and edaphic conditions. Norway spruce is shade-tolerant and well-adapted to cool, moist climate but is challenged in drier and warmer conditions, whereas Sessile oak is light-demanding and well-suited to temperate climates with moderate rainfall and well-drained soils. European beech, which requires moderate light for optimal growth, is climatically between the two species, well-suited to temperate climates with consistent moisture and moderate temperatures but challenged in regions experiencing increased temperatures or prolonged droughts.

To maintain analytical consistency, we exclusively utilized increment cores from even-aged, monospecific stands. This restriction minimizes assumptions related to interspecific competition and growth dynamics. Furthermore, to reduce confounding factors, we selected stands with no evidence of thinning or active management. This study is predicated on the following assumptions:

- Stand productivity is the primary driver of tree growth;
- Monospecific stands within the same ecoregion exhibit similar growth patterns;
- Unthinned monospecific stands display comparable growth trajectories.

These assumptions, formalized above, are assessed using diameter growth, estimated from ring width measurements. The first assumption prioritizes environmental influences over individual tree genetics, while the second emphasizes regional homogeneity within species ranges. The last assumption highlights the significance of human intervention on diameter growth, as stated by Eichorn [20] and Assmann [21].

### 2.1. Romanian NFI Dendrochronological Data

**Figure 1:**
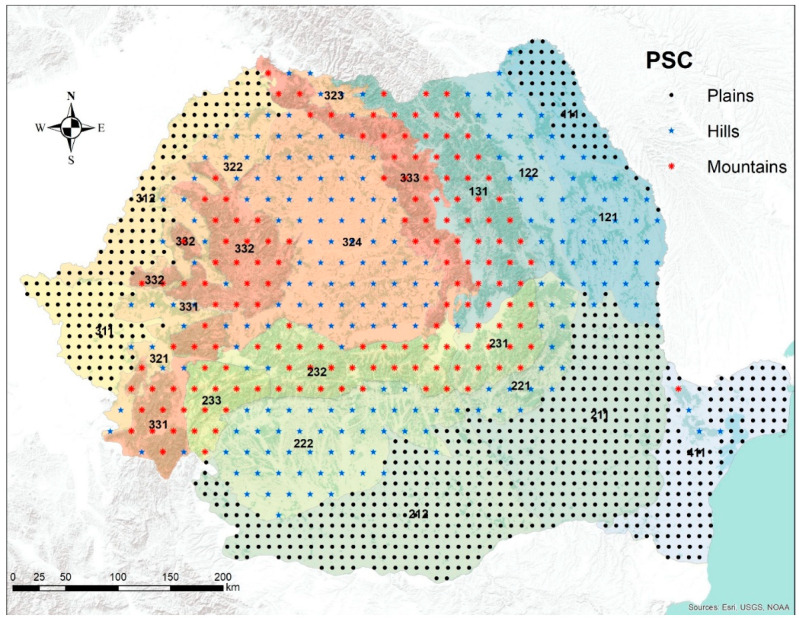
Layout of the Romanian National Forest Inventory. The shading represents elevation, darker being higher, and the background color delineates the ecoregion listed on Table 1. The density of the PSC is for visualization only. The numbers represent the ecoregion codes of Romania. The natural range of the tree species considered in our study covers 15 ecoregions.

The Romanian National Forest Inventory (NFI) employs a systematic sampling design utilizing permanent sample clusters (PSCs). Each PSC consists of four circular permanent sample plots (PSPs) positioned at the vertices of a 250 m square. These plots are measured every five years using a two-phase approach. Initially, aerial images are interpreted following the methodology of Paine and Kiser [22], which serve as input for the second phase that consists in terrestrial measurements. The Romanian NFI employs a systematic sampling design based on a 4 km × 4 km quadratic grid (Figure 1). Each grid cell is subdivided into 16 squares of 1 km × 1 km. A permanent sample cluster (PSC) is located in the southwest corner of each 1 km × 1 km square within the 4 km × 4 km grid. To ensure adequate sampling coverage across Romania’s major geomorphological regions—plains, hills, and mountains—the 4 km × 4 km grid is densified to a 2 km × 2 km grid in the plains region, where forest cover is less dense (Figure 1). The Romanian NFI comprises a total of 31,201 PSCs and 124,804 permanent sample plots (PSPs)

For each permanent sample plot (PSP), species, and cohort, an average of two increment cores were extracted at breast height (1.3 m). However, in PSPs with only one species and cohort, three to four cores were collected. Increment cores were sampled from randomly selected dominant and codominant trees within each PSP. The detailed extraction procedure is described in Marin et al. [12]. While over 50,000 increment cores were collected and processed, this study focused on a subset of 6536 cores, originating from 1655 PSCs. The limited number of cores used reflects the study’s focus on three species within even-aged, monospecific stands. The ring width of each increment core was measured with the graphical procedure of Lebourgeois and Merian [23] based on the images produced by the high resolution scanner Epson XL1000. (Epson Ltd., Akita, Japan) Each increment core was cross-dated using the standard procedures [24,25,26,27,28]. The selected 6536 increment cores contain 427,635 rings, the majority belonging to European beech (i.e., 241,240), followed by Norway spruce (136,904) and Sessile oak (49,491). For each ring, five attributes were recorded: ecoregion, species, year, age (measured in years), and width (measured in micrometers). Following the ecoregion definition of Omernik and Griffith [29], Romania’s territory was delineated into 21 regions. However, the tree species of interest in this study were not found in every ecoregion.

To reduce the chance of including outlier values of DBH growth because of special environmental conditions, we have included in analysis only increment cores from trees within the natural distribution range, which restricted the Norway spruce only to the mountainous regions, the European beech to the hill and mountainous regions, and the Sessile oak to the hill regions. The age constraint, combined with the requirements of pure species unmanaged stands and natural range distribution led to the analysis of only 15 ecoregions: seven for Norway spruce, 15 for European beach, and eight for Sessile oaks (Table 1).

**Figure 2:**
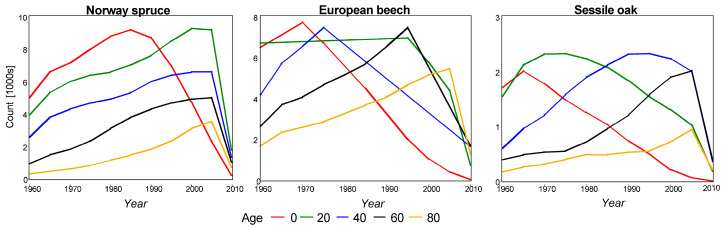
Number of rings for each species from 1960 to 2010.

To avoid inclusion in the analysis of periods with a very different climate than today, we considered only rings less than 100 years old. Therefore, the earliest year when the ring accumulated was 1860. The selection of mono-species unmanaged stands did not impact the power of the analysis, as sufficient rings were available every year (Figure 2). The maximum age of a ring for all species and all years was 99, except for Sessile oak, which exhibited a decrease from 2000, but only for ages less than 5 years.

### 2.2. Romanian Climate Data

According to the Koppen Classification system, Romania has a temperate–continental climate that is spotted by alpine climate and cool continental [30]. The country’s climate is dominated by three regional centers, one located on the Mediterranean areas, one on the Scandinavian–Baltic area, and one over the Russian steppe. Romania has an extensive network of meteorological stations, relatively even distributed across the country, out of which 188 measure precipitation, 150 relative humidity, air temperature, and pressure, and 127 soil temperature [31].

To effectively combine the wide spatial coverage of the NFI network with climate data, meteorological variables must be extrapolated. One of the most accurate and precise weather descriptions across Romania was developed by Dumitrescu and Birsan [31], called ROCADA, which predicts nine meteorological variables with a spatial resolution of 0.1° (approximately 11.1 km at 45° north). The nine variables are air pressure, minimum, maximum, and average air temperature, soil temperature, precipitation, sunshine hours, cloud cover, and relative humidity. ROCADA provides accurate, high-resolution extrapolations for warm-season variables like summer and spring temperatures and precipitation, which are important for tree growth. However, its lower accuracy during winter months indicates that its estimates should primarily be used for warm-season analyses.

**Figure 3:**
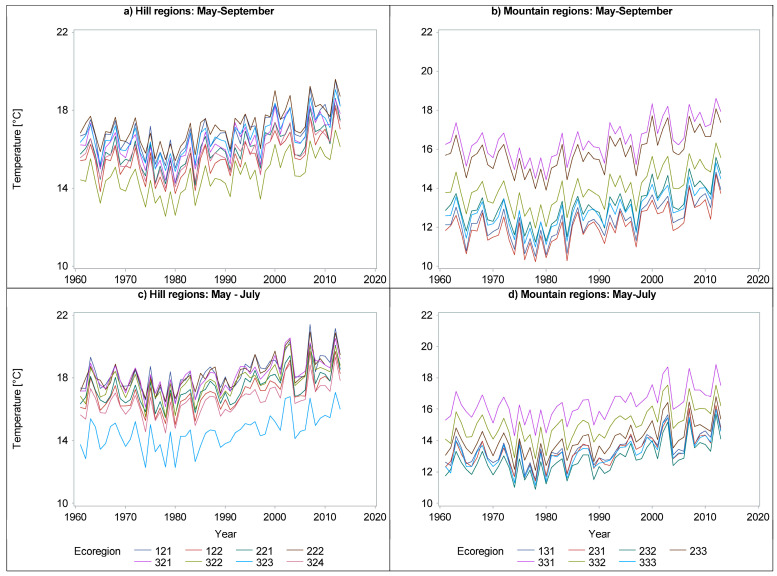
Temperatures of the warmer and wetter months (i.e., May–September -> (**a**,**b**)) or only warmer months (i.e., May–July -> (**c**,**d**)) according to the geomorphology of the ecoregions (i.e., hills (**a**,**c**) and mountains (**b**,**d**)).

To examine the relationships between ecoregions, climate, and diameter growth and yield, we utilized all nine variables from ROCADA, supplemented by four additional climate variables hypothesized to influence radial growth. These variables were (1) average temperature from May to July, (2) average temperature from May to September, (3) cumulative precipitation from May to July, and (4) cumulative precipitation from May to September. Temperature was selected due to its fundamental role in biological processes, with the warm-season average (May to September) representing a critical period for tree growth (Figure 3a,b). To account for the drier conditions in August and early September, we added a variable that specifically considered the precipitation and temperature of the May to July period (Figure 3c,d).

**Figure 4:**
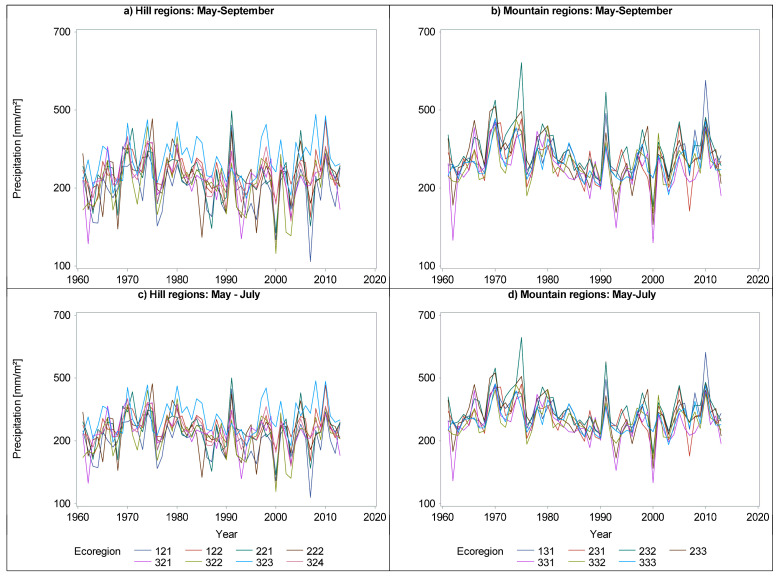
Precipitation for the warmer and wetter months (i.e., May–September -> (**a**,**b**)) or only warmer months (i.e., May–July -> (**c**,**d**)) according to the geomorphology of the ecoregions (i.e., hills (**a**,**c**) and mountains (**b**,**d**)).

Mirroring the temperature, we computed the cumulative precipitation for the wetter and warmer months, as well as for the warmer ones (Figure 4). Different from temperature, the precipitation experienced a minute change in August and September for both hill and mountains regions (Figure 4b,d vs. Figure 4a,c, respectively).

### 2.3. Assessment of the Climate Impact on DBH Dynamics

**Figure 5:**
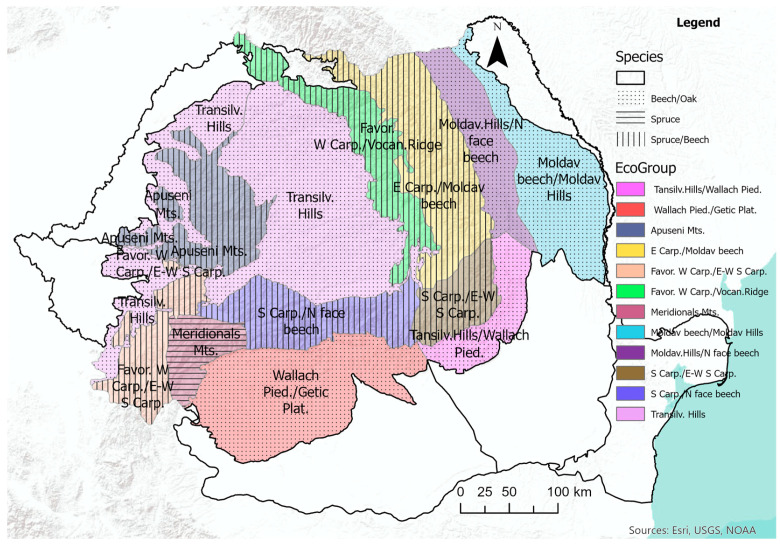
Consolidation according to Table 2 of ecoregions into ecogroups for each species. The shade shows elevation, with darker color being higher.

Marin et al. [4] proved that the radial growth for the three species considered in this study exhibits similar patterns among several ecoregions. Therefore, the assessment of climate impact on DBH growth was executed on groups of ecoregions, subsequently referred to as ecogroups, rather than individual ecoregions (Table 2). Consequently, for Norway spruce we focused only on four ecogroups (Figure 5), six ecogroups for European beech, and five ecogroups for Sessile oak.

**Figure 6:**
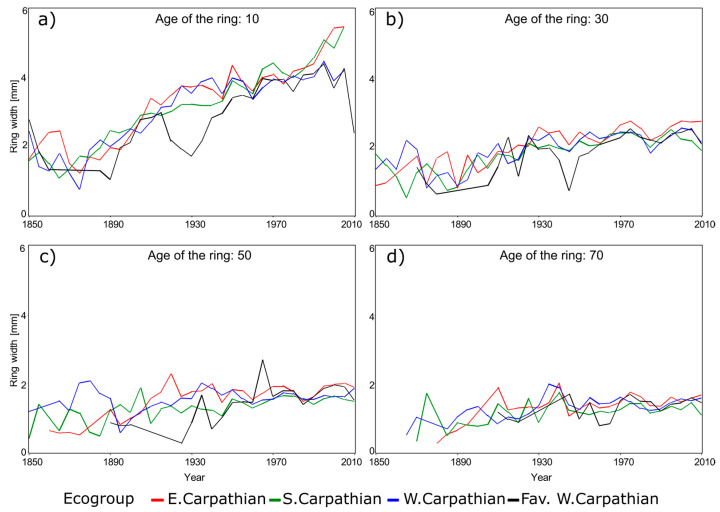
Temporal radial growth for Norway spruce at selected ages: (**a**) 10 years (**b**) 30 years (**c**) 50 years (**d**) 70 years.

**Figure 7:**
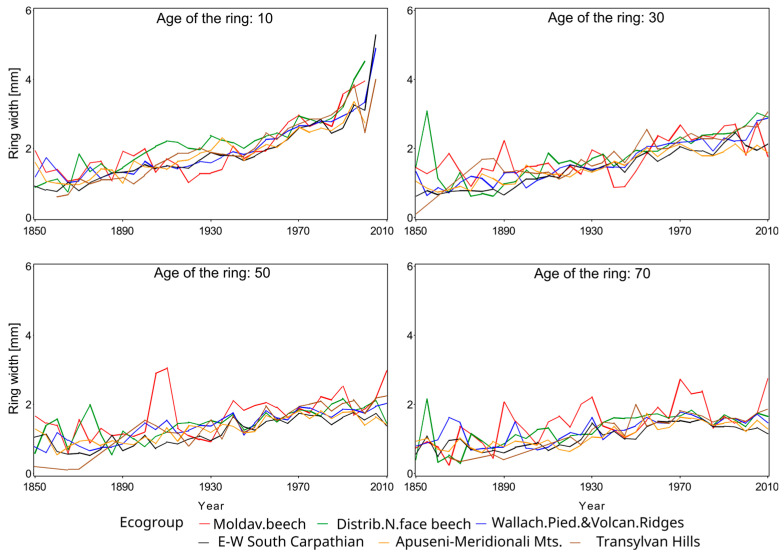
Temporal radial growth for European beech at selected ages.

**Figure 8:**
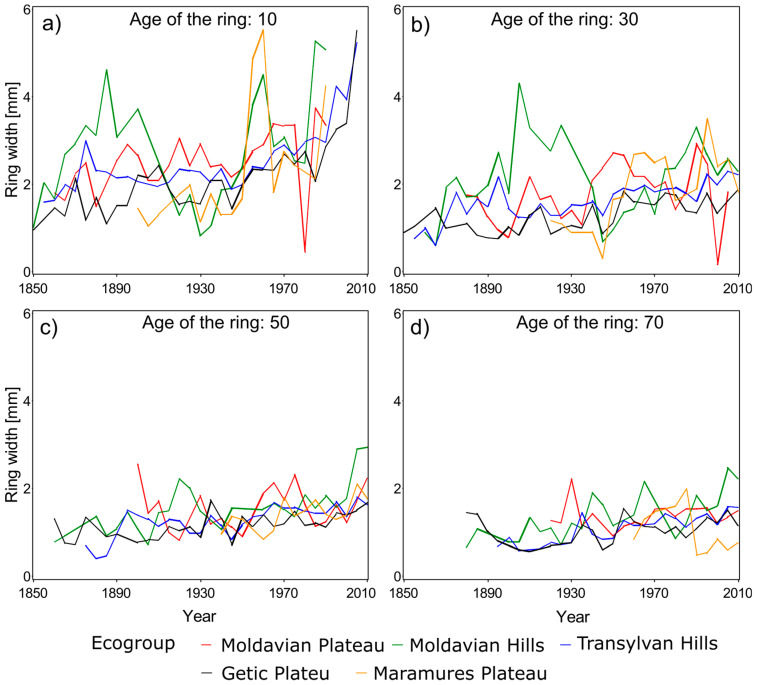
Temporal radial growth for Sessile oak at selected ages: (**a**) 10 years (**b**) 30 years (**c**) 50 years (**d**) 70 years.

A visual inspection of temperature and precipitation in the last 60 years (Figure 6, Figure 7 and Figure 8) suggests a possible change in the dynamics of DBH based on the climate. A plethora of studies comparing climate variables with dimensional and age variables revealed statistically significant impacts on tree growth and yield, demonstrating the sensitivity of forest productivity to climatic fluctuations [32,33].

To assess the impact of climate on stem’s cross section we initially focused on the relationship between dimensional attributes (i.e., growth and yield of radius and basal area) and ecogroup, year, and age (Equation (1)). We considered the age to account for the temporal dynamics of each species and the time to incorporate the environmental change. We used linear discriminant analysis for repeated measures [34,35], with a covariance matrix that has an autoregressive of order 1 structure, as suggested by Lix and Sajobi [36]:

(1) d=ecogroups+year+age+e

where *d* is the diameter-based variable of interest, namely ring width, DBH, BA increment, or BA

*year* is the calendar year of the variable *d*
*age* is the age, in years, of the variable *d*
*e* is the error

Figure 3 demonstrates a clear upward trend in temperature over the past 45 years. Consequently, we hypothesized that climatic factors significantly influence tree diameter growth. To quantify this relationship, for species where our baseline model (Equation (1)) was statistically significant, we incorporated nine climate variables from the ROCADA dataset. To maintain model parsimony and facilitate interpretation, we initially employed a linear model (Equation (2)). However, if climate variables failed to exhibit significant linear relationships with radial growth, then despite observed trends in both radial dynamics and ROCADA variables, we explored nonlinear model formulations.

(2) $$d=ecogroups+year+age+\sum_{i}Climate\;Variable_{i}+e$$

Regardless of the linear model used Equation (1) or (2), we assessed the normality of residual distributions using the Kolmogorov–Smirnov and Anderson–Darling tests. In cases where normality was rejected, we visually inspected residual distributions, focusing on mode, skewness, and kurtosis. Following Glass et al. [37], we accepted inference based on normality for unimodal, mildly skewed distributions. However, for multimodal or severely skewed distributions, alternative model formulations were explored.

The autocorrelation of the residual was evaluated using the Durbin–Watson test [38,39]. When significant autocorrelation was detected, indicating a lack of residual independence, Equation (1) or (2) were refitted to account for temporal dependencies, as determined by the model developed based on Equation (3).

(3) $$e_{t}=e_{t-1}+e_{t-2}+e_{t-3}+e_{t-4}+e_{t-5}+\varepsilon_{t}$$

where *e_t–i_* is the *ith*-lag error as produced by the repeated measurements linear model at year *t*

*ε_t_* is the error at year *t*

The analytical phase was considered complete when the error term *ε_t_* exhibited white noise properties [34,40,41]. The white noise properties were tested using the same tests as before, with Kolmogorov–Smirnov and Anderson–Darling for normality and Durbin–Watson for independence. In the eventuality that *ε* is not normally distributed but is unimodal, exhibits reduced skewness, and has no temporal interdependencies, we concluded that the models can be used for inference, even though not as efficient as expected. We executed all the analysis in SAS 9.4 [42], with a significance level of 0.01.

## 3. Results

### 3.1. Data Inspection

Radial growth displayed a general increasing trend across all species (Figure 6, Figure 7 and Figure 8), although the magnitude of this trend varied among ecoregions and age classes (e.g., Figure 6a vs. Figure 6d or Figure 8b vs. Figure 8d). This variability underscores the necessity of regionalized models [4]. The extensive combination of ecoregions, species, years, and ages resulted in missing data for older stands in some instances (e.g., Figure 6c or Figure 8d).

Radial growth trends show a temporal increase that coincides with a documented temperature rise in Romania, approximately 1 °C per decade since 1980 (Figure 3). This warming trend mirrors the global increase in atmospheric carbon dioxide concentrations, as indicated by the National Oceanic and Atmospheric Administration [43]. However, data from certain Romanian ground stations suggest potential regional variations [44].

### 3.2. Repeated Measures Analysis Without Climate Attributes

We found that ecoregion significantly influenced radial growth across all species and age classes from 5 to 60 years (Table 3 presents 10-year increments for brevity). Repeated measures analysis corroborated the visual trends observed in Figure 6, Figure 7 and Figure 8. However, the inferential power of this analysis was reduced by violations of homoscedasticity and normality assumptions (Levene’s test and Kolmogorov–Smirnov test, *p* < 0.01). Despite these violations, the residual distributions exhibited leptokurtosis, unimodality, and limited skewness, suggesting reasonable inferential validity, albeit with reduced efficiency [37]. Conversely, the Durbin–Watson test revealed significant residual autocorrelation (*p* < 0.001 for all ages), raising concerns about the robustness of the findings. Only at age 50, for Norway spruce and Sessile oak, did residuals exhibit white noise. To address this autocorrelation, we implemented autoregressive models, ranging from one to four terms depending on species and age, with younger ages generally requiring more terms (Table 3). This autoregressive correction effectively eliminated residual correlation, yielding Durbin–Watson test *p*-values > 0.2 for all ages, thus validating the robustness of our findings.

The significant impact of time on radial growth suggests that climate could be one of the factors contributing to the increase in DBH, as temperature also changed in the last 30 years. Similarly to radial growth without climate, we found that irrespective of the species, ecogroup, age, and year significantly affected the change in DBH (Table 4 and Table 5), expressed either as growth (i.e., DBH or BA growth) or cumulatively (i.e., DBH or BA). When climate was included in the analysis, among all the nine variables from ROCADA and a multitude of their combinations, we found that only cumulative precipitation from May to July, the average temperature during the growing season (May–September), and the average temperature during the wet growing season (May–July) have an impact on DBH change (Table 4 and Table 5). Depending on the perspective on the DBH change, we found that precipitation during May–July affects only the growth and not the yield, regardless of the attribute type, namely linear (i.e., DBH) or quadratic (i.e., BA). Different from precipitation, which influences only the growth and not the yield, the temperature impacts both the growth and the yield, but in a nuanced way. First, temperature does not affect the Norway spruce and European beech yield, just the growth, and not even for all attributes, as the BA growth for Norway spruce seems to be unaffected by temperature. Second, not the same temperature seems to influence the species and attributes, as May to September averages govern the Norway spruce and European beech yields (i.e., DBH and BA), and May to July shapes the growth and yield of DBH for Sessile oak.

### 3.3. Repeated Measures Analysis with Climate Attributes

Similarly to the analysis without climate, irrespective of the attributes and species, the residuals were not normally distributed and heteroskedastic (Levene’s test for homogeneity of variance and the Kolmogorov–Smirnov and Anderson–Darling normality tests had *p*-values < 0.001). Considering that all the relationships were linear, it was not a surprise to notice that the residuals were leptokurtic, unimodal, and without significant skewness. Therefore, the inference lacks efficiency but is still correct if the independence assumption is fulfilled. Unfortunately, the Durbin–Watson test revealed significant correlations among the residuals, regardless of the species and attributes. To remove the temporal autocorrelation, we used an autoregressive model, which depending on the species and attributes, has between one and five lag terms (Table 4). The models for growth present between four and five lag terms, whereas almost all the yield models have one term, except for the BA of Norway spruce, which has four terms (Table 5).

The inclusion of the climate variables in the analysis provided a more distilled perspective on the ecoregions that exhibit similar DBH and BA changes. For Norway spruce, all four attributes considered in the study (i.e., DBH growth and yield and BA growth and yield) were separated into two areas (i.e., (1)) Eastern and Southern Carpathians and (2)) Western Carpathians). For European beech, the mountain ecoregions were also separated into two groups irrespective of the attributes, namely (1) Eastern Carpathians and (2) Eastern and Southern Transylvanian ridges. Sessile oak has a very different behavior than Norway spruce and European beach, as it has three groups of ecoregions for DBH and four groups for BA. Furthermore, the grouping within each attribute was different, as the tree groups for DBH growth were (1) Moldavian piedmonts, (2) Getic, Transylvanian, and Maramures plateaus, and (3) Cris Hills, and for DBH yield were (1) Moldavian plateau, (2) Moldavian Hills, and (3) Southern Carpathians piedmonts, Transylvanian and Maramures plateaus, and Cris Hills. For BA, the four groups of ecoregions were (1) Moldavian piedmonts, (2) Southern Carpathian piedmonts and Maramures plateau, (3) Cris Hills, and (4) Transilvania plateau for the growth and (1) Moldavian plateau, (2) Moldavian Hills, (3) Southern Carpathians piedmonts, Cris hills, and Transylvanian plateau, and (4) Banat Mountain, Transylvania, and Maramures plateaus for the yield. Irrespective of the species and attributes, the difference between the groups of ecoregions exhibited a *p*-value < 0.001.

## 4. Discussion

The main finding of our study is that the consideration of spatiality in quantifying tree dynamics is mandatory but not necessarily sufficient, as climate could contribute to significant alteration of the growth of individual trees. This major result holds across all three species, even though they have different climatic and edaphic requirements. While the response varies among the species, the inclusion of some measures of temperature or precipitation is recommended. Radial growth increased over time across all species (Figure 6, Figure 7 and Figure 8), suggesting environmental changes within the past 50 years, potentially related to soil or climate. While ROCADA’s high-resolution interpolated precipitation data showed minimal change, mean annual temperature increased by approximately 1 °C per decade. The observed biennial precipitation pattern, despite minimal overall change, and the temperature increase may be attributed to land use changes impacting weather station environments, particularly urban expansion (e.g., the transformation of the Baneasa station).

The temperature increase significantly influenced DBH and BA growth for all species. Although precipitation changes were less pronounced, they also impacted growth. Conversely, DBH and BA yield were insensitive to precipitation and, except for Sessile oak, to temperature. This lack of sensitivity in yield, coupled with the minimal precipitation change and the smoothing effect of yield computation, suggests that long-term water availability does not significantly affect stem dimension accumulation.

Residual autocorrelation indicated that growth was influenced by up to five previous years for European beech and four for Norway spruce and Sessile oak. While the autoregressive models used to address this autocorrelation may have confounded climatic influences, they highlighted the importance of time and past growth in addition to ecoregion and age. Notably, growth was influenced by at least four preceding years.

The ecoregion classifications of Marin et al. [4] were refined by incorporating climatic variables. Norway spruce growth differences were primarily attributable to climate, with western regions exhibiting milder (oceanic, sub-Mediterranean) climates and eastern/southern regions experiencing more extreme (arid, Scandinavian–Baltic) conditions [45]. This climatic differentiation held across all attribute and measurement types. Similar climatic influences were observed for European beech, with Eastern Carpathian populations exposed to cold Eurasian steppe winds and Transylvanian populations experiencing milder conditions due to protection from the Meridional Mountains. For both species, climate enhanced ecoregion delineation compared to spatial distribution alone [4], suggesting climate’s dominant role in development over geomorphology and pedology. Among the climate attributes, the most influential was the temperature during the May–September.

Sessile oak exhibited a more nuanced climatic response. It was the only species with yield sensitivity to temperature, and different temperatures influenced linear (DBH) and quadratic (BA) measures of across-stem dimensions. As a warm-adapted species, Sessile oak’s heightened sensitivity to temperature and precipitation was expected in a rapidly warming climate. The differential temperature influence on linear and quadratic measures likely reflects the cumulative nature of BA yield, which contrasts with radial growth. The May–September temperature integrates the warmest months, while May–July represents the active growth period, given limited late-season precipitation.

Our study is constrained by its assumptions and inherent limitations. As such, the impacts of climate and regionalization on tree development can only be firmly stated for single-species stands of Norway spruce, European beech, or Sessile oak that have never been thinned. We assumed that site productivity, driven by edaphic and climatic factors, is the primary determinant of stand development, implying that changes in these methodological attributes would elicit a tree response. However, a range of factors not considered in this study could produce similar responses, such as increased CO_2_ availability due to proximity to human activities or changes in the genetic stock used for regeneration. The primary limitation of our study, which is also its strength, lies in its focus on unmanaged, single-species stands. While this approach provides clear and convincing evidence for mono-species stands, it does not represent the majority of forested land, which often consists of managed, complex, multi-species stands. Consequently, our findings cannot be generalized to such diverse forest systems.

This study supports the development of regional growth and yield models for Romanian tree species, incorporating both geomorphology and climate. The temporal growth trends necessitate the inclusion of a calendar year term. Addressing the challenge of establishing growth asymptotes may be achieved by incorporating temperature and precipitation data. Our study demonstrates the feasibility of integrating climatic variables into empirical models alongside traditional inventory attributes, enabling the derivation of environmental–production relationships typically associated with process-based models [46,47,48].

## 5. Conclusions

The accuracy of growth and yield models in predicting forest development is often compromised by local environmental variability. Moreover, climate dynamics introduces further uncertainty into site-specific estimates, even if overall model precision appears stable. The analysis of Romanian National Forest Inventory (NFI) data revealed a consistent increase in diameter growth since 1960, partially attributable to rising temperatures. Repeated measures analysis, corroborating visual assessments of radial changes in Romania’s three dominant tree species, identified significant inter-ecoregional differences, aligning with previous findings [4].

Employing climatic variables, we partitioned growth and yield into diameter at breast height (DBH) and basal area, providing a more detailed analysis than relying solely on annual ring width. Among nine climate attributes (sourced from ROCADA [31]), growing season temperature and total precipitation significantly influenced radial and basal area growth. However, precipitation showed no measurable impact on yield, and temperature significantly affected yield only in Sessile oak.

Beyond ecoregional and climatic factors, the calendar year significantly impacted DBH and basal area changes, indicating a temporal influence on growth and yield that extends beyond simple chronological time. This temporal effect encompasses tree age and past environmental conditions. Radial and basal area growth were significantly influenced by the preceding four to five years, while yield was affected only by the prior year. This suggests a long-term adaptive memory in trees.

Despite minor deviations from normality and homoscedasticity assumptions, the unimodal distribution and low skewness of residuals support the robustness of our results. The consistent responses across diverse geomorphological and climatic conditions in the three studied species reinforce the need for regional forest ecosystem assessments that incorporate past environmental conditions (up to five years) and key climate attributes.

Our study emphasizes the necessity of developing regional models for accurate growth and yield estimations. Specifically, incorporating growing season temperature and precipitation, alongside traditional forest inventory data, is crucial. The significant influence of the calendar year further supports its inclusion in such models. However, challenges remain in accurately estimating asymptotic values for DBH and basal area. Future research using process-based models could elucidate the magnitude of these asymptotes.

## Figures and Tables

**Table 1:** Number of incremental cores and annual rings across the 15 ecoregions on which the analysis was executed. The ecoregion code shows its location on the Romania map from Figure 1.

Ecoregion (Code)	Elevation	Number of Incremental Cores/Number of Rings		
	m	Norway Spruce	European Beech	Sessile Oak
Moldavian Plateau (121)	200–400		41/2881	93/6575
Moldavian Hills (122)	200–500		112/8687	29/2385
Eastern Carpathians (131)	800–2300	986/49,576	332/2286	
Buzau-Vrancea piedmonts (221)	200–800		142/11,572	87/4400
Getic plateau (222)	200–700		223/15,496	219/13,962
Buzau-Vrancea mountains (231)	800–1900	336/15,749	292/24,447	
East Southern Carpathians (232)	1000–2500	506/27,969	387/30,700	
West Southern Carpathians (233)	1000–2500	84/4259	258/20,280	
Caras Hills (321)	200–600		20/1577	17/1419
Cris Hills (322)	200–600		31/2379	44/2969
Maramures plateau (323)	300–800		4/272	11/706
Transilvania Plateau (324)	300–800		187/13,763	253/17,075
Banat Mountains (331)	800–1800	47/1654	427/36,007	
Western Carpathians (332)	800–1800	228/9102	285/21,448	
Volcanic ridge (333)	1000–2000	568/28,595	287/23,445	
Total		2755	3028	753

**Table 2:** Grouping of ecoregions that exhibits similar radial growth according to Marin et al. [4].

Norway Spruce		European Beech		Sessile Oak	
Ecogroup	Ecoregion	Ecogroup	Ecoregion	Ecogroup	Ecoregion
Eastern Carpathians	131	Moldavian beech	121 131	Moldavian Plateau	121
Southern Carpathians	231 232	Distributed north facing beech	122 232 323	Moldavian Hills	122
Western Carpathians	332	Transylvanian hills	321 322 324	Transylvanian hills	221 321 322, 324
Favorable Western Carpathians	233 331 333	Wallachian piedmonts and volcanic ridges	221 222 333	Getic plateau	222
		East–West Southern Carpathians	231 331	Maramures plateau	323
		Apuseni–Meridionali Mountains	233 332		

**Table 3:** Impact of the groups of ecoregions on radial growth. *** represents *p*-values < 0.001.

Species	Attribute	Age				
		10	20	30	40	50
Norway spruce	Ecogroup	***	***	***	***	***
	Year	***	***	***	***	***
	no. autoreg. terms	4	2	4	2	0
European beech	Ecogroup	***	***	***	***	***
	Year	***	***	***	***	***
	no. autoreg. terms	3	2	4	4	1
Sessile oak	Ecogroup	***	***	***	***	***
	Year	***	***	***	***	***
	no. autoreg. terms	2	2	2	1	0

**Table 4:** Impact of ecogroups, age, year, and climate on the growth in DBH and basal area. *** represents *p*-values < 0.001, ** represents *p*-values < 0.01.

Attribute	Species					
	Norway Spruce		European Beech		Sessile Oak	
	DBH	BA	DBH	BA	DBH	BA
Group of Ecoregions	***	***	***	***	***	***
Year	***	***	***	***	***	***
Age	***	***	***	***	***	***
Precipitation May–July	***	**	***	***	***	***
Temperature May–Jul	-	-	-	-	***	-
Temperature May–Sep	***	-	***	***	-	***
Independence	No	No	No	No	No	No
no.no. terms autoregression	4	4	5	5	4	4

**Table 5:** Impact of ecogroups, age, year, and climate on the yield (cumulative growth) in DBH and basal area. *** represents *p*-values < 0.001, * represents *p*-values < 0.05.

Attribute	Species					
	Norway Spruce		European Beech		Sessile Oak	
	DBH	BA	DBH	BA	DBH	BA
Group of Ecoregions	***	***	***	***	***	***
Year	***	***	***	***	***	***
Age	***	***	***	***	***	***
Temperature May–July	-	-	-	-	***	-
Temperature May–September	-	-	*	-	-	***
Independence	No	No	No	No	No	No
no. terms autoregression	1	4	1	1	1	1

## Data Availability

The data presented in this study are openly available in ScholarArchives@OSU at https://ir.library.oregonstate.edu/concern/datasets/8336h726s (accessed on 4 March 2025), DOI doi.org/10.7267/8336H726S.
